# Experiencia en el uso de los protocolos Biomed-2 para el estudio de reordenamientos de TCR e inmunoglobulinas en proliferaciones linfoides en el Instituto Nacional de Cancerología, Colombia

**DOI:** 10.7705/biomedica.5940

**Published:** 2022-05-01

**Authors:** Nicolás Villamizar-Rivera, Natalia Olaya

**Affiliations:** 1 Grupo de Patología Oncológica, Instituto Nacional de Cancerología, Bogotá, D.C., Colombia Instituto Nacional de Cancerología Bogotá, D.C. Colombia; 2 Maestría en Genética Humana, Facultad de Medicina, Universidad Nacional de Colombia, Bogotá, D.C., Colombia Universidad Nacional de Colombia Universidad Nacional de Colombia Bogotá, D.C. Colombia; 3 Facultad de Medicina, Universidad Nacional de Colombia, Bogotá, D.C., Colombia Universidad Nacional de Colombia Universidad Nacional de Colombia Bogotá, D.C. Colombia

**Keywords:** linfoma, reordenamiento génico de linfocito T, inmunoglobulinas, genes codificadores de los receptores de linfocitos T, electroforesis en gel de poliacrilamida., Lymphoma, gene rearrangement, T-lymphocyte, immunoglobulins, genes, T-cell receptor, electrophoresis, polyacrylamide gel.

## Abstract

**Introducción.:**

El consorcio europeo BIOMED-2 se creó para determinar si una población linfoide de difícil clasificación patológica es clonal. En Colombia, la implementación de estas pruebas comenzó en el 2015 en el Instituto Nacional de Cancerología E.S.E. (Bogotá).

**Objetivos.:**

Determinar el comportamiento de las pruebas de reordenamiento clonal o clonalidad linfoide. y determinar las dificultades de su uso en nuestro medio verificando su adaptación local y los resultados en una serie retrospectiva de casos y consecutiva de proliferaciones linfoides sometidas a los protocolos BIOMED-2.

**Materiales y métodos.:**

A partir de las historias clínicas, se recolectaron los datos clínicos e histológicos y los resultados de los análisis de los reordenamientos en todos los casos de proliferaciones linfoides sometidas a los protocolos BIOMED-2, entre febrero de 2015 y mayo de 2019.

**Resultados.:**

Se hallaron 132 casos, de los cuales 47 se clasificaron mediante los protocolos de Biomed-2 como hiperplasias linfoides reactivas, 62 como linfomas T, 19 como linfomas B y 3 como neoplasias linfoides de linaje no establecido. Solo en un caso falló la extracción de ADN. Según estos resultados, la mayor dificultad diagnóstica para el patólogo fue el análisis de los infiltrados linfoides T, la mayoría (44) de los cuales correspondía a lesiones cutáneas.

**Conclusiones.:**

Las pruebas de clonalidad pueden usarse en tejidos de diversa calidad en nuestro medio como ayuda en el diagnóstico de proliferaciones linfoides de difícil clasificación. Es importante hacerlas e interpretarlas de manera multidisciplinaria y considerar cada caso por separado.

## Abstract (en)

**Introduction:**

The European BIOMED-2 consortium was created to evaluate clonality in lymphoproliferations that are difficult to diagnose. In Colombia, the implementation of these tests began in 2015 at the *Instituto Nacional de Cancerología E.S.E.*, Bogotá.

**Objectives:**

To determine the behavior of the rearrangement tests for lymphoid clonality and the difficulties of its implementation in our field through a series of retrospective and consecutive cases of lymphoid proliferation subjected to the BIOMED-2 protocols.

**Materials and methods:**

Clinical and histological data and the results of the rearrangement analysis of all cases of lymphoid proliferation subjected to the BIOMED-2 protocols between February 2015 and May 2019 were collected from clinical histories.

**Results:**

We recovered 132 samples from which 47 corresponded to reactive lymphoid hyperplasias, 62 to T lymphomas, 19 to B lymphomas, and three to lymphoid neoplasms of unestablished lineage. Only in one case did DNA extraction fail. According to these results, the greatest diagnostic difficulty for the pathologist was the analysis of T lymphoid infiltrates, most of which (44) were skin lesions.

**Conclusions:**

Clonality tests can be used in tissues of different quality to help in the diagnosis of lymphoid proliferations that are difficult to classify. It is important to implement and interpret them in an interdisciplinary way considering each case separately.

Los linfomas son neoplasias malignas que surgen de cualquier célula del linaje linfoide y representan cerca del 10 % de los cánceres registrados en el Instituto Nacional de Cancerología ^1^^,^^2^ donde, en el 2015, se registraron 588 nuevos casos de tumores de los tejidos hematopoyético y linfoide ^3^. El diagnóstico de estas enfermedades se basa en la correlación de los hallazgos clínicos, la morfología de las células, la inmunohistoquímica, la citometría de flujo ^4^ y las pruebas de biología molecular ^5^.

Según van Dongen, *et al*. ^6^, incluso en centros de alto nivel, alrededor del 15 % de las proliferaciones linfoides no puede diagnosticarse con estas herramientas. Al parecer, este valor es una estimación de los autores, pues no hay estudios que determinen cuál es el grado de dificultad que los hematopatólogos enfrentan para clasificar una muestra como maligna o benigna.

En cada linfocito la recombinación somática elabora un exón que codifica para la región variable a partir de la selección de un segmento variable (V), un segmento de unión (J) y un segmento de diversidad (D), así como de la adición de nucleótidos N y P que posteriormente se transcriben en ARNm y, finalmente, se traducen y maduran en una proteína funcional (figura 1). El segmento D solo está presente en los lo de las cadenas pesadas de Ig, en tanto que los *loci* β y δ son exclusivos de las del TCR. La recombinación alélica ocurre cuando se unen los fragmentos variables de diversidad y unión, la llamada recombinación V(D)J, que se regula por medio de exclusión alélica; una vez que un alelo se ha reordenado, se envía una señal al otro para interrumpir el proceso ^7^.

**Figura 1:**
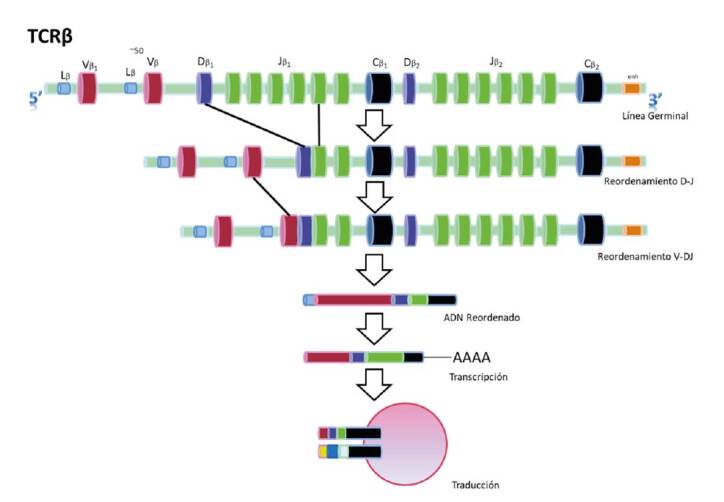
Proceso de reordenamiento del gen del receptor de antígeno

Se muestra el reordenamiento de la cadena beta del receptor de células T (TCR), proceso que es muy similar para las demás cadenas del TCR y para las inmunoglobulinas. En este proceso se pueden llegar a tener hasta 10^18^ combinaciones.

La reacción en cadena de la polimerasa (PCR) para amplificar los segmentos reordenados de la región variable (V), de diversidad (D) y de unión (J) de los genes de las inmunoglobulinas (Ig) y del receptor de células T (TCR) permite discriminar, en general, si una población celular corresponde a un proceso reactivo (policlonal) o es una neoplasia maligna (monoclonal) ^8^. En la mayoría de los casos, los linfomas de células B evidencian reordenamientos de las Ig, mientras que los linfomas de precursores de células T reflejan rearreglos en el TCR ^9^. Sin embargo, hay excepciones a la correlación entre la malignidad y el reordenamiento clonal o clonalidad de estos genes; por ejemplo, gracias al proceso de recombinación somática, algunos linfomas maduros del centro germinal pueden mostrar reordenamientos policlonales. De igual manera, algunas lesiones reactivas, especialmente las de origen autoinmune, pueden presentar reordenamientos clónales ^10^.

Hasta el 2003, el análisis de estos reordenamientos se hacía mediante *Southern blot*, método que requería grandes cantidades de ADN ^11^. Posteriormente, se desarrollaron protocolos basados en la PCR que buscaban aumentar la sensibilidad de la prueba, pero se obtenían falsos negativos ^12^^,^^13^. En ese mismo año, se conformó un consorcio europeo de laboratorios denominado BIOMED-2 (actualmente EuroClonality), el cual estandarizó los protocolos para detectar la clonalidad tanto de Ig como de TCR mediante PCR multiplex ^14^.

Los protocolos permitieron obtener mayor sensibilidad, especificidad y reproducibilidad para detectar el reordenamiento clonal en neoplasias linfoides ^15^^-^^17^. Además, se elaboraron guías para la correcta interpretación que también dan cuenta de posibles inconvenientes ^18^. Esta metodología se ha convertido en el estándar para determinar la clonalidad linfoide ^6^ y es la base de los protocolos de biología molecular para evaluar la enfermedad mínima residual linfoide propuestos por el consorcio europeo EuroMRD ^19^.

Los laboratorios del mundo han adaptado la técnica a diferentes tipos de tejido, entre ellos, biopsias decalcificadas de médula ósea ^20^, tejidos fijados en formalina y embebidos en parafina (FFEP), biopsias de piel ^21^ y biopsias por aspiración con aguja fina ^22^. Los resultados han sido variables y, según las conclusiones de los estudios, lo ideal es utilizar tejido fresco. Las enfermedades en las cuales se ha usado la prueba de clonalidad incluyen la leucemia linfoblástica aguda de precursores B ^23^, el linfoma Hodgkin clásico ^24^, el linfoma folicular ^25^, las linfoproliferaciones cutáneas ^26^, los linfomas anaplásicos de células T ^27^ y las micosis fungoides ^28^, entre otras.

El objetivo del presente estudio fue describir el comportamiento de los reordenamientos del receptor de antígeno en una serie consecutiva de proliferaciones linfoides de difícil clasificación, en pacientes del Instituto Nacional de Cancerología en Bogotá, mediante una adaptación local de los protocolos BIOMED-2.

## Materiales y métodos

### Obtención de los datos

Se analizaron todos los casos consecutivos de proliferaciones linfoides atípicas para las cuales se solicitó el estudio de reordenamientos del receptor de antígeno, entre febrero de 2015 y junio de 2019. Se examinaron las historias clínicas y se elaboró una base de datos con los datos clínicos, histológicos e inmunohistoquímicos. En la base de datos de laboratorio, se examinaron las características de las muestras recibidas para estudio, la calidad y concentración de ADN, y los resultados de clonalidad.

### Indicaciones para el estudio de clonalidad linfoide

La prueba se hacía por solicitud de los hematooncólogos, dermatólogos oncólogos y patólogos, en aquellos casos en que no había sido posible llegar a un consenso acerca del potencial maligno de la proliferación linfoide con base en los estudios de morfología, inmunohistoquímica, genética y citometría de flujo.

### Preparación de los tejidos

*Médula ósea y sangre.* La separación de leucocitos se hizo por gradiente de densidad utilizando el Histopaque 1077™ (Sigma-Aldrich, St. Louis, Missouri, USA).

*Tejido fresco.* El tejido se embebió en resina de congelación Tissue Freezing Medium (Leica biosystems, Wetzlar, Alemania), se congeló y se hicieron cortes representativos de un grosor de 3 µm para luego utilizar la coloración de rutina y evaluar el porcentaje de células problemáticas y su distribución.

*Tejido fijado en formalina y embebido en para fina.* Se evaluaron las láminas de hematoxilina y eosina de cada caso y se seleccionaron aquellas con más del 10 % de población linfoide problemática; cuando este porcentaje era menor, se sugería tomar una nueva biopsia y procesarla en fresco.

### Extracción de ADN

En los tejidos fijados en formalina y embebidos en parafina, se hicieron cuatro cortes de 10 pm del bloque y se colocaron en un tubo Eppendorft de 1,5 ml; la desparafinación se hizo con 1 ml de xileno y 1 mi de etanol, repitiendo dos veces cada paso. Las células separadas por gradiente de densidad, el tejido fresco y el recién desparafinado, se dejaron en digestión en proteinasa K con una solución tampón de incubación (SDS al 2 %, 250 mM de NaCI, 1 mM de EDTA, 1 mM de Tris) durante 48 horas a 56 °C y, luego, se adicionaron perclorato de sodio (3M) y cloroformo y alcohol isoamílico en proporción de 24:1 (Sigma Aldrich, San Luis, Misuri, Estados Unidos); se mezcló homogéneamente y se centrifugó.

A la fase acuosa se le adicionó una sal fuerte (NaCI 3M) y etanol para precipitar el ADN y se mantuvo a 4 °C durante una hora; después, se centrifugó y el precipitado se lavó con etanol al 100 % y al 70 % para, finalmente, diluir el ADN en solución tampón de dilución (Tris 10 mM, EDTA 1 mM).

### Selección de blancos

Según el consorcio Biomed-2/EuroClonality, los tejidos en parafina se deben evaluar teniendo en cuenta la calidad del tejido, lo que permite evitar falsos negativos o errores en la amplificación. Además, los protocolos deben estandarizarse según los medios de cada centro diagnóstico. Como la prueba es costosa para los estándares nacionales y no está contemplada en el Plan Obligatorio de Salud, cada vez que se solicita debe tenerse en cuenta la pregunta clínica y la premura con la cual el médico tratante requiere los resultados. Por ello, deben elegirse con precisión los grupos de genes que se van a utilizar y los tamaños esperados.

### Reacción en cadena de la polimerasa

**Figura 2:**
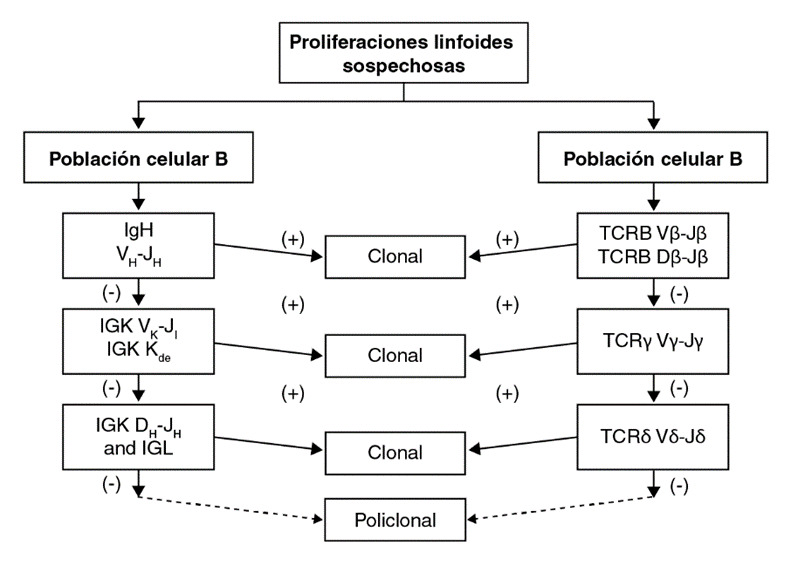
Algoritmo propuesto y utilizado para el estudio de clonalidad en lesiones linfoides

El ADN se cuantificó en el equipo Nanodrop 2000c™ (Thermo-Fisher, Waltham, Massachusetts, USA), utilizando una concentración final de 20 ng/ ul. La PCR múltiplex se ajustó a los protocolos diseñados en el consorcio BIOMED-2 ^29^, utilizando el IdentiClone lGH + IGK&TCRB + TCRG Gene Clonality Assay™ (Invivoscribe, San Diego, California, USA). La calidad de los tejidos en parafina se evaluó utilizando la PCR de control incluida en el mismo estuche. Se asumió que todos los tejidos en fresco eran de óptima calidad. Todas las PCR para amplificar el TCR y las inmunoglobulinas, se hicieron por duplicado y los productos se analizaron por electroforesis en gel de acrilamida o por análisis de fragmentos en secuenciador capilar según las recomendaciones del consorcio EuroClonality/Biomed-2 (figura 2) y la disponibilidad ^14^.

Los reordenamientos se evalúan de acuerdo con el origen celular de la lesión. Si los resultados son positivos, la muestra se clasifica como clonal. En caso contrario, se utilizan los siguientes reordenamientos sugeridos por flechas consecutivas, como se muestra en la figura. Si ningún resultado es positivo, la muestra se considera policlonal.

### Electroforesis en gel de acrilamida

Para favorecer la formación de heterodupletas, primero se usó el calentamiento y luego un choque térmico a 0 °C para después hacer la migración en geles TBE Novex™ al 6 % (Life Technologies, Invitrogen, Grand Island, NY, USA), utilizando la solución tampón de corrido BlueJuice Gel Loading Buffer™ (10X) (Thermo-Fisher, Waltham, Massachusetts, Estados Unidos) en una relación de 1:3 con el producto de PCR. En cada montaje se utilizó como referente el marcador de peso VC 100 bp Plus DNA Ladder™ (Vivantis, Selangor Darul Ehsan, Malaysia). El corrido se realizó en una cámara de electroforesis XCell SureLock Mini-Cell Electrophoresis System™ (ThermoFisher, Waltham, Massachusetts, Estados Unidos), a 110 v constantes durante 80 minutos y se reveló sumergiendo los geles en bromuro de etidio diluido (0,5 µ/1 ml) en agua destilada durante 10 minutos y, posteriormente, en agua de grado de biología molecular dos veces durante 10 minutos cada vez. Los geles se revelaron en el equipo Gel Doc XR+ Gel Documentation System™ (Bio Rad, Hercules, California, USA) y se tomó registro fotográfico de cada uno.

### Análisis de fragmentos

Como ya se mencionó, se formaron heterodupletas por calentamiento y luego se estabilizaron con un choque término a 0°C. Se hizo un duplicado de cada prueba de PCR y se visualizó en un analizador genético ABI PRISM 310™.

Se mezclaron 15 µl de Hi-Di Formamide™ (Applied Biosystems, Hampton, New Hampshire, USA), 0,4 µ de GeneScan 500 ROX Dye Size Standard™ (Applied Biosystems, Hampton, New Hampshire, USA) y 1 µ del producto de la PCR de cada muestra.

El corrido estándar con POP-6 POP-6 Polymer™ (Applied Biosystems, Hampton, New Hampshire, USA) se hizo en un capilar de 47 cm. Cada muestra se corrió durante 50 minutos para evitar errores en la lectura, y cada blanco se analizó individualmente comparándolo con el control clonal y el policlonal.

### Interpretación de los resultados

Los resultados fueron examinados independientemente por el biólogo molecular y el médico patólogo. Todas las muestras que presentaron una banda o pico definido en el rango establecido para cada tubo, se consideraron como clónales para ese blanco; las muestras que no mostraban un producto específico, en las que solo se veía un barrido de ADN o una distribución de tipo campana de Gauss, se consideraron policlonales. Cuando las bandas eran inespecíficas o no concluyentes, se repetían la PCR y la electroforesis bajo las mismas condiciones. Cada caso se interpretó con base en los hallazgos clínicos y anatomopatológicos, y se reportó siguiendo las recomendaciones de Langerak, *et al*. ^6^.

## Resultados

### Pacientes y muestras

Se evaluaron 122 pacientes, 58 de ellos mujeres con edades entre los 12 y los 90 años (52,25%) y 64 hombres, entre los 7 y los 94 años (52,09 %). De algunos se obtuvieron varias muestras de tejidos diferentes. Se evaluaron 142 muestras; de una de estas no fue posible obtener suficiente ADN y, en otras, no se obtuvo el ADN y tampoco se pudo tomar una nueva muestra (0,7 %), por lo que se procesaron 131 muestras en total.

### La prueba permitió el uso de cualquier tipo de tejido.

Se utilizaron todos los tipos de tejido, pero principalmente, los fijados en formalina y embebidos en parafina (n=81; 61,83 %). También, se procesaron tejidos no parafinados de biopsias de piel en solución salina (n=31; 23,66 %), de sangre periférica (n=11; 8,39 %), de aspirado de líquido de médula ósea (n=5; 3,81 %), de ganglio en solución salina (n=2; 1,52 %) y de hueso (n=1; 0,76 %). Los tejidos fijados en formalina y embebidos en parafina que se utilizaron tenían menos de un año de procesados; en promedio, se obtuvieron 152,6 ng/µl de ADN con 1,81 de pureza, en tanto que, de los tejidos no parafinados, se obtuvieron 294,2 ng/µl de ADN con 1,93 de pureza. El ADN extraído de los tejidos en parafina amplificó por lo menos 300 pb en la PCR de control desarrollada con el mismo protocolo de BIOMED-2, es decir, con una calidad que permitió evaluar la mayoría de los reordenamientos sin error de falsos negativos.

### La prueba de clonalidad permitió establecer el diagnóstico patológico.

El diagnóstico final de los casos después de que los médicos tratantes y los patólogos conocieran el resultado de los reordenamientos, correspondió a 19 proliferaciones de células B (12,5 %), 62 proliferaciones de células T (43,75 %), tres proliferaciones de linaje incierto (2,68 %) y 47 hiperplasias reactivas (35,87 %). De las 47 hiperplasias reactivas, 46 fueron linfoides (95,8 %) y una correspondió a prurigo (4,2 %). En tres casos analizados para confirmar las proliferaciones de células T, se vieron reordenamientos pseudoclonales y, en algunas hiperplasias linfoides reactivas, se observó una distribución irregular de los picos en el análisis de fragmentos, problema que se solucionó analizando más de los reordenamientos disponibles para lograr un análisis concluyente.

**Figura 3:**
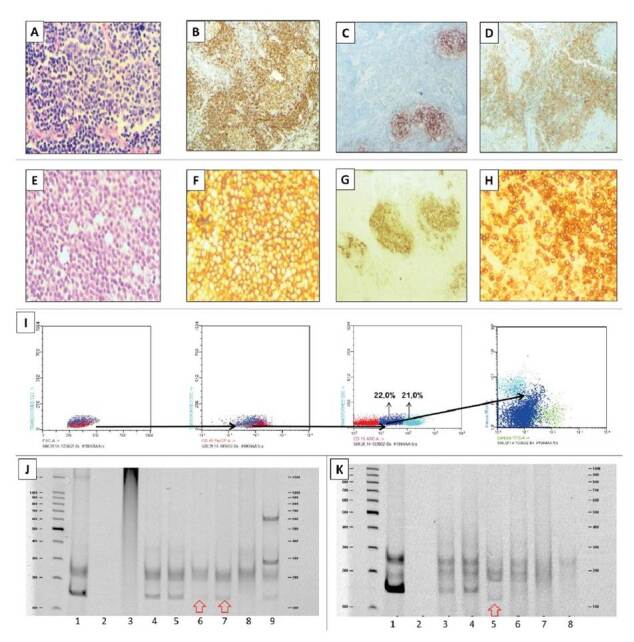
Caso representativo del diagnóstico multidisciplinario en casos de clonalidad

Según la pregunta clínica y la calidad del ADN, se eligieron los reordenamientos que podrían contribuir al diagnóstico. En el caso que se presenta en la figura 3, una paciente con antecedente de linfoma cutáneo presentó linfadenopatías y, mediante el análisis de reordenamientos en la piel y el ganglio linfático, se resolvió la duda clínica de si se trataba de la misma proliferación linfoide o de otra completamente diferente (figura 3).

Mujer de 69 años con lesiones en ganglio linfático e infiltrado en la piel. El equipo clínico quería determinar si eran lesiones de diferente origen. A. Lesión de piel, hematoxilina y eosina 400X. B. CD79a, 100X C. CD23, 100X D. BCL2, 100X. E. Biopsia de ganglio linfático, hematoxilina y eosina 400X. F. CD25, 100X. G. CD23, 100X. H. BCL2, 100X. I. Citometría de flujo que revela una población celular irregular. J. Electroforesis en gel de poliacrilamida para los reordenamientos de IgK, carril 1, control positivo; 2, control negativo; 3, control policlonal; carril 6: biopsia de piel (clonal); carril 7: biopsia de ganglio linfático (clonal). K. Reordenamientos de la región FR3 de IgH; carril 1, control positivo; 2, control negativo; 3, control policlonal; carril 5: biopsia de piel (clonal); carril 6: biopsia de ganglio linfático (policlonal)

**Figura 4:**
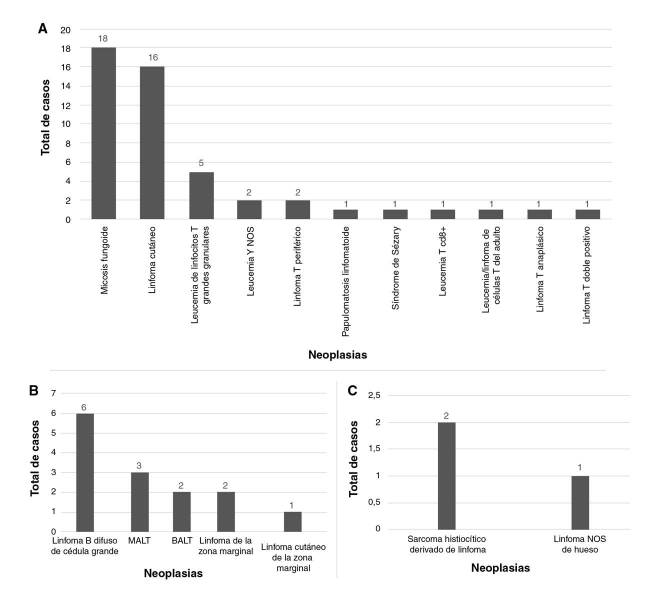
Número total de neoplasias diagnosticadas con la prueba de clonalidad, 2015-2019

*Clonalidad en proliferaciones de células B.* Se diagnosticaron 19 linfomas de células B, 6 de alto grado (linfomas B difusos de células grandes) y 13 de bajo grado (asociados con cualquier tejido, los primarios cutáneos de la zona marginal, los de la zona marginal, la alteración linfoproliferativa asociada con inmunodeficiencia primarla de tipo polimorfo con rearreglo clonal de IGH, el linfoma no Hodgkin primario del sistema nervioso central asociado con inmunodeficiencia y la leucemia B NOS (*Not Otherwise Specified*) (figura 4B).

A. Proliferaciones de linfocitosT. B. Proliferaciones de linfocitos B. Proliferaciones analizadas entre el 2015 y el 2019. A. Proliferaciones de linfocitos T. B. Proliferaciones de linfocitos B. C. Proliferaciones de origen celular indeterminado.. MALT: *Mucosa-Associat*ed *Lymphoid Tissue*. BALT*: Bronchus Associated Lymphoid Tissue*

**Figura 5:**
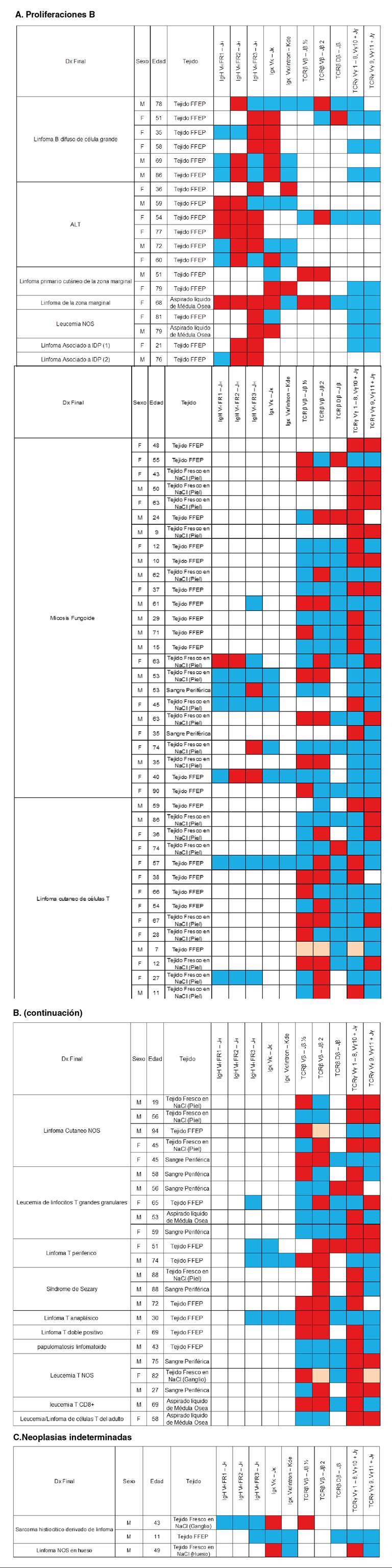
Distribución de los reordenamientos del receptor de antígeno en cada una de las proliferaciones analizadas

En los linfomas asociados con cualquier tejido (BALT y MALT), se analizaron los reordenamientos de las cadenas pesadas de las Ig y se observó que IgH VH - FR2 - JH se reordenó en todos los cinco casos analizados, en tanto que VH - FR1 - JH solo se reordenó en tres y, VH - FR3 - JH, en cuatro. Solamente en uno de los casos analizados, se reordenó IgK. Además, en este último caso, también se reordenó TCRp Vp - jp 2 (figura 5).

Reordenamientos del receptor de antígeno para cada una de las proliferaciones analizadas. La columna correspondiente a "linfoma asociado a IDP "^1^ correspondió a una alteración linfoproliferativa asociada con inmunodeficiencia primaria, con rearreglo clonal de IGH y la llamada " linfoma asociado con IDP" ^2^: linfoma no Hodgkin primario del sistema nervioso central asociado con inmunodeficiencia primaria.

En un linfoma de la zona marginal y en uno cutáneo de la zona marginal, se analizaron los reordenamientos de TCRβ Vβ - Jβ ½ & 2, los cuales fueron clónales. El linfoma de la zona marginal, la leucemia B NOS y los linfomas asociados con alguna inmunodeficiencia primaria, mostraron reordenamientos, por lo menos, en una región de IgH.

La distribución de los reordenamientos clonales de las inmunoglobulinas para las neoplasias de células B, fue la siguiente: 40 % para IgH VH FR1 - JH (4/10), 92 % para IgH VH FR2 - JH (11/12), 70 % para IgH VH FR3 - JH (12/17), 64 % para Igκ Vκ - Jκ (9/14) y 22 % para Igκ Vκ/intron - Kde (2/9). En el caso de los reordenamientos de TCR, los resultados fueron de 50 % para TCRβ Vβ - Jβ ½ (2/4), 50 % para TCRβ Vβ - Jβ 2 (4/5), 25 % para TCRβDβ - Jβ (1/4), 0 % para TCRγ Vγ 1 - 8, Vγ10 + Jγ (0/10) y 0 % para TCRγ Vγ 9, Vγ11 + Jγ (0/9) (figura 3).

*Clonalidad en proliferaciones de células T.* Se analizaron 63 linfomas de células T, de los cuales 44 correspondieron a linfomas cutáneos (micosis fungoides, linfomas cutáneos de células T y linfomas cutáneos NOS) y 18 a linfomas de otro origen (leucemia de linfocitos T grandes granulares, linfoma T periférico, síndrome de Sézary, linfoma T anaplásico, linfoma T doble positivo, papulomatosis linfomatoide, leucemia T NOS, leucemia T CD8+ y leucemia/linfoma de células T del adulto) (figura 4B). 

De los linfomas cutáneos se analizaron 25 micosis fungoide, 14 linfomas cutáneos de células T y 5 linfomas cutáneos NOS. En total, los reordenamientos en los linfomas se distribuyeron así: 54 % (21/44) para TCRγVγ 1 - 8, Vγ10 + Jγ, 47 % (18/38) para TCRβ Vβ - Jβ 2, 44 % (16/36) para TCRβ Vβ - Jβ ½, 40 % (17/42) para TCRγ Vγ 9, Vγ11 + Jγ y 12 % (3/25) TCRβ Dβ - Jβ. Además, hubo reordenamientos en el 33 % (3/9) para IgH VH FR3 - JH, el 28 % (2/7) para IgH VH FR2 - JH, el 14 % (1/7) para IgH VH FR1 - JH, y no se observaron reordenamientos de Igk en ninguna de estas neoplasias (figura 5). 

En los linfomas no cutáneos hubo reordenamientos para TCRγ Vγ 1 - 8, Vγ10 + Jγ en 83 % (15/18), para TCRβ Vβ - Jβ 2 en 55 % (10/18), para TCRβ Vβ - Jβ ½ en 46 % (7/15), para TCRγ Vγ 9, Vγ11 + Jγ en 37 % (6/16) y para TCRβ Dβ - Jβ en 18 % (2/11). No se evidenció ningún reordenamiento para Ig (figura 5).

Los reordenamientos clonales del TCR para las neoplasias T se distribuyeron así: 44 % (23/52) para TCRβ Vβ - Jβ ½, 50 % (28/56) para TCRβ Vβ - Jβ 2, 13 % (5/36) para TCRβ Dβ - Jβ, 58 % (36/62) para TCRγ Vγ1 - 8, Vγ10 + Jγ y 39 % (23/58) para TCRγ Vγ 9, Vγ11 + Jγ. En el caso de los reordenamientos de Ig, fueron: 14 % (1/7) para IgH VH FR1 - JH, 28 % (2/7) para IgH VH FR2 - JH, 23 % para IgH VH FR3 - JH (3/13,), ninguno (0/9) para Igκ Vκ - Jκ y ninguno (0/4) para Igκ Vκ/intron - Kde (figura 5).

*Clonalidad en proliferaciones de linajes indeterminados*. Las proliferaciones indeterminadas correspondieron: a dos sarcomas histiocíticos derivados de linfoma (uno sin ningún reordenamiento y otro con el de Igκ Vκ- Jκ y TCRβ Vβ - Jβ ½) y a un sarcoma NOS de hueso (con reordenamiento de Igκ Vκ - Jκ y de TCRγ Vγ 1 - 8, Vγ10 + Jγ).

## Discusión

Esta es la primera experiencia en Colombia en el uso de reordenamientos del receptor de antígeno para determinar la clonalidad linfoide. Se describe una serie consecutiva de proliferaciones linfoides de diagnóstico difícil, en la cual se utilizaron los protocolos BIOMED-2 para evaluar los reordenamientos que ocurren en las cadenas pesadas y ligeras de las inmunoglobulinas, y en las cadenas beta y gamma del receptor de células T.

Los protocolos BIOMED-2 utilizados en el estudio se consideran actualmente la prueba de referencia para establecer si una proliferación es clonal, pues superan la inmunofenotipificación ^30^. Aunque este análisis es posible por secuenciación de nueva generación, está todavía se encuentra en validación, por lo cual el protocolo de mayor utilización en el mundo sigue siendo el de la PCR múltiplex.

La adaptación de la prueba es sólida y permite utilizar todo tipo de tejidos; fue especialmente exitosa con los parafinados, de los cuales se desconocen muchas veces las condiciones preanalíticas por ser el Instituto Nacional de Cancerología un centro de referencia nacional. Atribuimos los buenos resultados a nuestro protocolo para la extracción orgánica de ADN.

Los resultados mostraron que los linfomas cutáneos y los linfomas B difusos de células grandes son las proliferaciones linfoides que los patólogos consideran más difíciles de diagnosticar y clasificar. En general, los reordenamientos obtenidos para cada lesión son los esperados según la literatura especializada ^27^^,^^31^^,^^32^.

Un hallazgo interesante fue la presencia de reordenamientos del TCR en linfomas B difusos de células grandes, en los de la zona marginal y en los sarcomas histiocíticos, lo que se explicaría por dos razones principales: los linfocitos acompañantes del microambiente tumoral son clones de linfocitos T o las características aberrantes de las células tumorales permiten el reordenamiento de los genes del TCR sin necesidad de expresarlos ^27^^,^^33^. Se requieren nuevos estudios para aclarar estos mecanismos. Por esta razón, cuando se considere utilizar la prueba para determinar el linaje, siempre deben interpretarse sus resultados con precaución.

Las pruebas para evaluar reordenamientos en linfoproliferaciones sospechosas desarrolladas por el consorcio BIOMED-2/EuroClonality, son sólidas y permiten el análisis diagnóstico de casos difíciles en la mayoría de los casos y en varios tipos de tejido, incluidos los parafinados y los de hueso. Esto es importante en nuestro medio, donde no se controlan con precisión las condiciones preanalíticas.

Los análisis de reordenamientos fueron útiles para el diagnóstico, pero no para establecer el linaje de la población celular problema, pues se encontró que los reordenamientos del TCR pueden aparecer en proliferaciones linfoides B y, los de inmunoglobulinas, en proliferaciones linfoides de células T. Es necesario el análisis multidisciplinario caso por caso, con el fin de optimizar el uso de reactivos y responder adecuadamente a cada una de las necesidades clínicas.
